# Stabilization of Li_0.33_La_0.55_TiO_3_ Solid Electrolyte Interphase Layer and Enhancement of Cycling Performance of LiNi_0.5_Co_0.3_Mn_0.2_O_2_ Battery Cathode with Buffer Layer

**DOI:** 10.3390/nano11040989

**Published:** 2021-04-12

**Authors:** Feihu Tan, Hua An, Ning Li, Jun Du, Zhengchun Peng

**Affiliations:** 1 Key Laboratory of Optoelectronic Devices and Systems of Ministry of Education and Guangdong Province, College of Physics and Optoelectronic Engineering, Shenzhen University, Shenzhen 518060, China; tanfeihu@163.com (F.T.); huaan@szu.edu.cn (H.A.); ln5858518@hotmail.com (N.L.); 2 School of Microelectronics, South University of Science and Technology, Shenzhen 518055, China; dujun@grinm.com

**Keywords:** thin films, all-solid-state batteries, interface, buffer layer

## Abstract

All-solid-state batteries (ASSBs) are attractive for energy storage, mainly because introducing solid-state electrolytes significantly improves the battery performance in terms of safety, energy density, process compatibility, etc., compared with liquid electrolytes. However, the ionic conductivity of the solid-state electrolyte and the interface between the electrolyte and the electrode are two key factors that limit the performance of ASSBs. In this work, we investigated the structure of a Li_0.33_La_0.55_TiO_3_ (LLTO) thin-film solid electrolyte and the influence of different interfaces between LLTO electrolytes and electrodes on battery performance. The maximum ionic conductivity of the LLTO was 7.78 × 10^−5^ S/cm. Introducing a buffer layer could drastically improve the battery charging and discharging performance and cycle stability. Amorphous SiO_2_ allowed good physical contact with the electrode and the electrolyte, reduced the interface resistance, and improved the rate characteristics of the battery. The battery with the optimized interface could achieve 30C current output, and its capacity was 27.7% of the initial state after 1000 cycles. We achieved excellent performance and high stability by applying the dense amorphous SiO_2_ buffer layer, which indicates a promising strategy for the development of ASSBs.

## 1. Introduction

Over the past several decades, the successful application of lithium-ion batteries has revolutionized personal electronic devices and significantly changed our lifestyle [1,2]. All-solid-state lithium batteries (ASSBs) are promising battery systems for electric vehicles and smart devices owing to their safety, energy density, packaging, and operating temperature range compared with traditional liquid electrolytics [3,4,5]. However, the low ionic conductivity of solid electrolytes hinders their commercial applications [6,7,8]. Organic solid electrolytes are characterized by good processability and high Li+ conductivity at relatively high temperatures [9,10] and are potentially used in large-scale applications such as wearable [11] and micro intelligent devices [12]. However, the volume power density and compatibility of the electrode with organic solid electrolytes are still limited [13,14]. Hence, inorganic solid electrolytes are potential candidates for applications in ASSBs. Many systems such as LiLaZrO_3_ [15], Li_2_S-P_2_S_5_ [16], and Li_2_NH [17] have been studied [18]. Among them, Li_x_La_y_TiO_3_ (LLTO) with a perovskite structure shows high ionic conductivity (10^−3^–10^−6^ S/cm) [19], good mechanical strength, and flexibility. However, the ionic conductivity of LLTO thin films is much lower than that of bulk LLTO [20], possibly because of the structural and phase transition from bulk to the thin film. Zhao et al. reported LLTO thin films prepared by magnetron sputtering with an ionic conductivity of 5.25 × 10^−5^ S/cm [21]. Son et al. prepared a film with an ionic conductivity of 9.51 × 10^−4^ S/cm by Y doping using the sol-gel method [22].

The interface between the solid-state electrolytes and the electrodes plays an important role in the performance of solid-state batteries [23,24]. Electrochemical reactions in ASSBs occur at the solid/solid contacting interface between the electrode and solid electrolyte, which differs from that in conventional batteries with liquid electrolytes. Thus, the formation of intimate contacts at the solid/solid electrode–electrolyte interfaces is a key to improve the electrochemical performance of ASSBs [25]. Surface modification techniques and introducing a buffer layer have proven effective in forming intimate interfaces and enhancing contact areas between the electrode and the electrolyte [26,27,28]. Pan et al. improved the Li-ion transport across the electrolyte/electrode interface by the MOF+ infiltration electrolyte [29], and the infiltration of the liquid electrolyte can improve the contact between the electrolyte and electrode. Al_2_O_3_ prevents the formation of harmful ion-neutral NiO with rock-salt structure at the interface; therefore, Su et al. used the atomic layer deposition (ALD) method to deposit an Al_2_O_3_ buffer layer of approximately 5 nm at the interface [30], thus improving the cycling performance of the battery.

Since the performance of the battery follows the “Cannikin law”, the interface between the solid electrolyte and the cathode or anode is just as important for improving battery performance. Numerous studies have focused on the interface between solid electrolyte and anode [4]. On the other hand, a high chemically stable interface with low-impedance between a solid electrolyte and cathode active materials remains a key challenge and is also highly desired for the development of ASSBs [31]. In this study, we have developed three types of buffer layers between cathode and solid electrolyte, then discussed their influence on the battery performance. Our results demonstrated that the buffer layers effectively improved the rate characteristics and cycling stability of the battery, and the sample with a SiO_2_ buffer layer could be cycled over 1000 times.

## 2. Materials and Methods

To study the electrolyte properties, we used an Ag/LLTO/Ag sandwich blocking electrode structure. The Ag electrode and the (Li_0.33_La_0.55_TiO_3_) LLTO thin film were prepared by magnetron sputtering (JGP-800, Zhongke Instrument Inc., Beijing, China). Sputtering power density was 2.63 W/cm^2^, and the sputtering gas was argon, target materials such as Ag and LLTO are from Kaistar (Kaistar electro-optic material Inc., Wuxi, Jiangsu, China). The LLTO film was annealed at different temperatures for 30 min at a heating rate of 10 °C/s using the RTP-800 type rapid annealing furnace.

Detailed experiments are as follows: LLTO thin films were deposited on the current collector Ag by radio frequency (RF) magnetron sputtering. The Ag films were deposited on LLTO, and the shape of each layer was controlled by a hard mask. The diameter of the LLTO film was approximately 3–4 mm, and the thickness was 300–400 nm. The sputtering power and argon pressure of LLTO films have been optimized and are kept constant to reduce interference factors. The effect of annealing on the solid electrolyte performance was investigated in the current study. After deposition, LLTO thin films were annealed at 300 or 400 °C for 30 min at a heating rate of 10 °C/s, and then cooled to 25 °C in a furnace. To evaluate the operational temperature range of solid electrolytes, we studied the sample properties between 25 and 120 °C in the variable temperature chamber.

A LiNi_0.5_Co_0.3_Mn_0.2_O_2_ (NCM) system with a high specific capacity was selected as the cathode and Li_0.33_La_0.55_TiO_3_ (LLTO) as the solid electrolyte. Li was chosen as the anode material. The buffer layer and the electrolyte were sputtered sequentially on the cathode. Three different buffer layers were deposited on the cathode and the electrolyte was deposited on the buffer layer. If the buffer layer is compatible with the chemical composition of the electrode and the electrolyte, it is called homogeneous. When the deposited buffer layer comprises new chemical components, it is called heterogeneous. There were four types of cells including the reference sample without the transition layer: NCM/LLTO/Li, NCM/(LLTO/NCM)/LLTO/Li, NCM/SiO_2_/LLTO/Li, and NCM/LiPON/LLTO/Li (Table 1). All buffer layers were prepared by sputtering. The homogeneous buffer layer is prepared as follows: First, the LLTO film is deposited on the NCM, and then the NCM film is deposited on the LLTO. The operation is repeated once to obtain a buffer layer with four layers. The power densities of NCM and LLTO were both 2.63 W/cm^2^ and the total deposition time was 40 min. SiO_2_ was deposited on NCM with a deposition power density of 2.19 W/cm^2^ for 20 min in argon. The deposition power density of the LiPON thin film was 2.19 W/cm^2^ in nitrogen. For electrochemical measurements, CR2032-type coin cells were assembled in a glovebox under a dry argon atmosphere (moisture and oxygen levels of less than 1 ppm).

Electrochemical impedance spectroscopy (EIS) measurements of the solid electrolyte were carried out on a CHI660E impedance analyzer with an amplitude voltage of 5 mV and frequency between 10 MHz and 100 kHz. The performance of the solid electrolyte was evaluated between 25 °C and 120 °C in a small temperature-controlled chamber. X-ray diffraction (XRD) patterns were collected using X’Pert PRO diffractometer (Philips Inc., Amsterdam, Netherlands), and the morphology and microstructures of the thin film were observed by field-emission scanning electron microscopy (FESEM, Nova NanoSEM 450, 5 kV, FEI Inc., Hillsboro, OR, USA) and transmission electron microscope (TEM, FEI Titan G2-300, 300 kV, FEI Inc., Hillsboro, OR, USA). The cells were cycled at a constant current between 3.2 (2.8 V) and 4.2 V versus Li/Li+ using a Keithley 2450 data source table (Tektronix Inc., Johnstown, OH, USA).

## 3. Results

### 3.1. Li_x_La_y_TiO_3_ (LLTO) Solid Electrolyte

**Figure 1:**
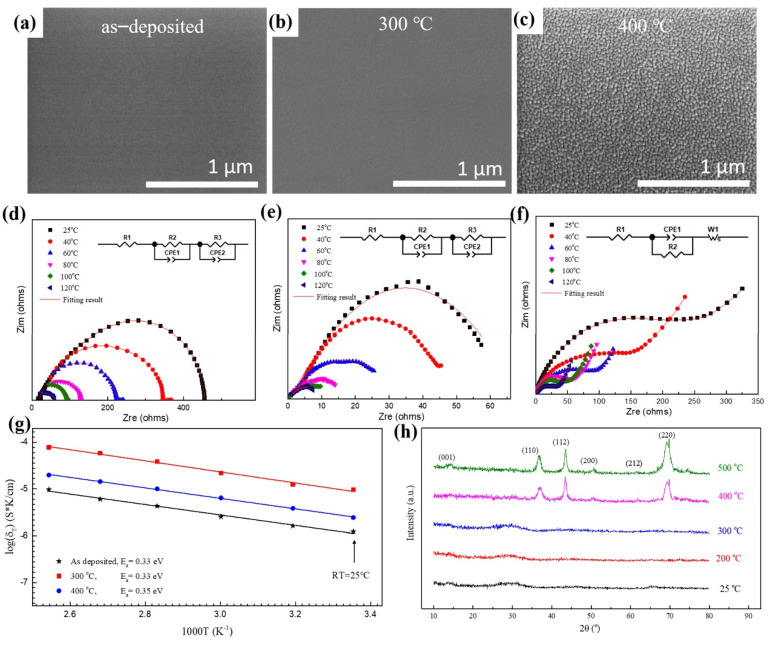
Microstructure and electrochemical properties of LLTO solid electrolyte thin films. Plane-view scanning electron microscopy (SEM) images of LLTO thin film annealed at different temperature: (**a**) as−deposited, (**b**) 300 °C, (**c**) 400 °C. Electrochemical impedance spectroscopy (EIS) measurements of the LLTO annealed at different temperature (inset is the equivalent circuit diagram): (**d**) as−deposited, (**e**) 300 °C, (**f**) 400 °C. (**g**) Arrhenius plots in the temperature range from 25 to 120 °C with an interval of 20 °C. (**h**) X-ray diffraction (XRD) data of LLTO thin films.

XRD patterns in Figure 1h indicate that both the as-deposited film and the film annealed at 300 °C were amorphous, and the samples crystallized presumably after annealing at 400 °C, which agrees with the scanning electron microscopy (SEM) images and electrochemical impedance spectra. The shape of the electrochemical impedance spectra suggests that there is no grain boundary resistance, since no additional semicircle is observed in Figure 1d,e, which is representative properties of grain boundary resistance. The average grain size for the LLTO film annealed at 400 °C was approximately 10–15 nm. Amorphous LLTO demonstrated high ionic conductivity, resulting from the lower migration potential of Li-ions in the amorphous layer than in the crystalline one. The insets in Figure 1d–f show the equivalent circuit diagrams obtained by fitting the electrochemical impedance spectra of the thin film. The fitting lines are in good agreement with the experimental data.

LLTO thin films exhibited good thermodynamic stability at the temperature between 25 and 120 °C, thus broadening the application temperature of batteries (Figure 1g). The ionic conductivity of LLTO increased with increasing temperature because it promotes the migration of lithium-ions in the electrolyte. The ionic conductivity of the film can be calculated from parameters such as thickness, area, and intrinsic resistance of the film (Figure 1g). The highest ionic conductivity obtained at room temperature and 120 °C was 9.56 × 10^−6^ S/cm and 7.78 × 10^−5^ S/cm, respectively. The ionic conductivity is similar to that of other perovskite and hydride solid electrolytes [23]. The activation energy of the LLTO thin film can be obtained from the Arrhenius plots in the temperature range from 25 to 120 °C (Figure 1g). The activation energy of the deposited film of 0.33 eV was obtained by fitting the curves of ionic conductivity and test temperature, and the activation energies of the films after heat treatment at 300 °C and 400 °C were 0.33 and 0.35 eV, respectively.

### 3.2. Microstructures of the Samples with Different Buffer Layers

**Figure 2:**
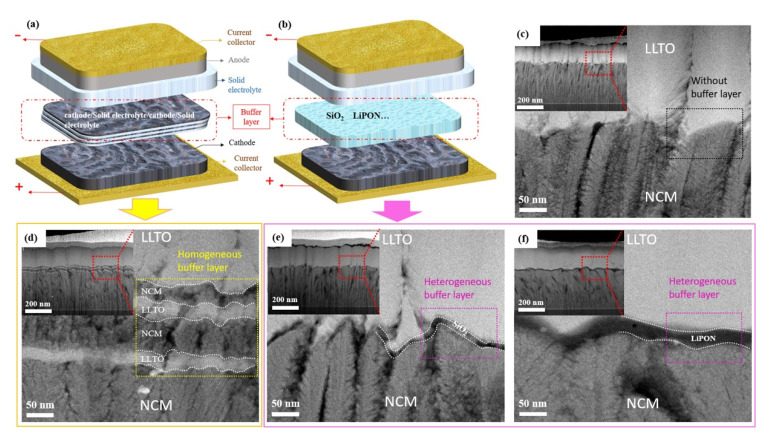
Schematic diagram of buffer layer: (**a**) Homogeneous buffer layer, (**b**) heterogeneous buffer layer. Cross-sectional transmission electron microscopy (TEM) image of NCM/LLTO thin-film interface, (**c**) NCM/LLTO, (**d**) NCM/(LLTO/NCM)/LLTO, (**e**) NCM/SiO_2_/LLTO, (**f**) NCM/LiPON/LLTO.

Based on the chemical composition of the buffer layer, we developed two types of transition layers and prepared a group of samples without a transition layer as a reference. Figure 2a,b show schematic diagrams of the homogeneous and heterogeneous buffer layers. Figure 2c shows the reference sample without a buffer layer. The homogeneous buffer was prepared by the co-sputtering of cathode and electrolyte materials (Figure 2d). The heterogeneous buffer layer was prepared from another material different from the electrode and electrolyte, for example, SiO_2_ and LiPON (Figure 2e,f).

Good contact between the electrolyte and the electrode can reduce the interfacial impedance of the battery and facilitate the migration of lithium-ions during charging and discharging. The gaps, holes, or other defects at the interface limit the effective contact area between the cathode and the electrolyte, thus reducing the speed of migration of lithium-ions. The microstructure and morphology of the interfaces between the electrode and electrolyte can be observed by cross-sectional TEM (Figure 2c–f). The thicknesses of the cathode (350–370 nm) and electrolyte (250–270 nm) for all samples remained identical because of the same preparation conditions and deposition time.

In the sample with homogeneous buffer layer (Figure 2d), we can observe that the interface presents a four-layer structure, and the composition of each layer from bottom to top is LLTO/NCM/LLTO/NCM. The interface is formed by the interleaving and overlapping of electrode and electrolyte, which can act as a buffer in the structure change between electrode and electrolyte, thus improving the bonding quality of the interface. In order to prevent the microstructure of the buffer layer such as SiO_2_ from being damaged during the sample preparation process, the cross section of the battery was prepared by focused ion beam (FIB), and then the morphology of the buffer layer was observed by high resolution TEM. The SiO_2_ and LiPON heterogeneous buffer layers with a thickness of 4–10 and 15–25 nm was deposited at the interface (Figure 2e and Figure 2f, respectively). Although the NCM film presents a bumpy structure and the thickness difference in some places is more than 50 nm, it can be seen from Figure 2e that the amorphous SiO_2_ changes along with the morphology of NCM. The SiO_2_ buffer layer formed a dense and smooth contact interface with the electrode and the electrolyte, resulting in an optimal interface with few defects.

### 3.3. Performance of the Batteries with Different Buffer Layer

#### 3.3.1. Rate Characteristics

**Figure 3:**
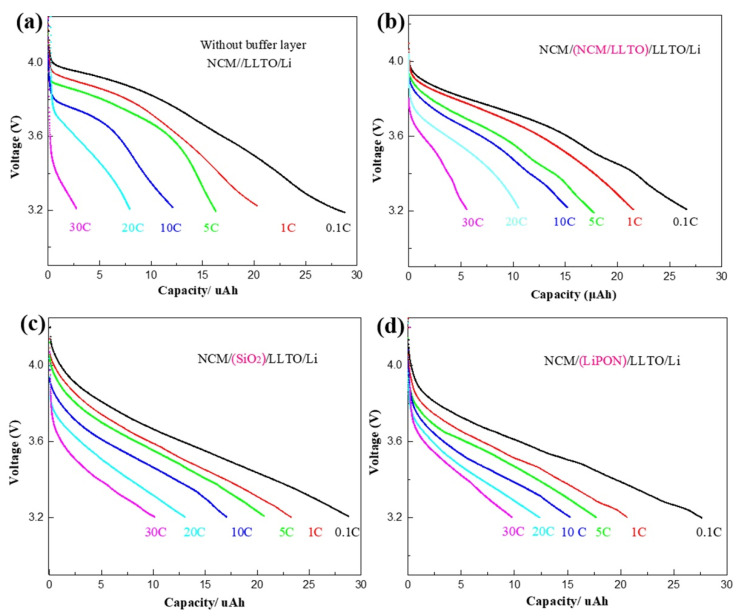
Rate characteristics of battery with different buffer layer: (**a**) NCM/LLTO, (**b**) NCM/(LLTO/NCM)/LLTO, (**c**) NCM/SiO_2_/LLTO, (**d**) NCM/LiPON/LLTO.

High-rate discharge characteristics are an important indicator of battery performance, which can reflect the high-power discharge performance of the battery. The rate characteristics of the samples with different buffer layers were tested at rates of 0.1, 1, 5, 10, 20, and 30 C, as shown in Figure 3a–d. Experimental data in this paper show that the capacity of the battery changes significantly during the first few dozen cycles, while the change tends to be slight after 50 cycles. Therefore, the rate characteristics of battery is tested after 50 cycles. Compared with the large effect of rate on capacity, the capacity change caused by “calendar life issues” is relatively small and can be ignored.

For all samples, the battery capacity and the discharge voltage platform decreased with an increase in the discharge rate. For the reference sample, the discharge rate increased from 0.1 C to 30 C, while the battery capacity decreased to 10% of the original value (Figure 3a). The rate characteristics of the samples with the transition layer were improved. In particular, when SiO_2_ heterogeneous buffer layer was introduced, the battery capacity at the discharge rate of 30 C can be maintained at 30% of that at the discharge rate of 0.1 C, as shown in Figure 3c.

The maximum discharge rate of a battery depends on the process where the lithium-ion migrates slowly. The diffusion rate of lithium-ions at the interface is the lowest, compared with the embedding rate at the electrode and the transmission rate in the solid electrolyte, and it is the rate-determining step [32]. D. Santhanagopalan et al. found that lithium-ions aggregated at the interface because of their low migration rate [33]. When the battery is discharged at a high rate, the lithium-ion migration rate at the interface is not high enough to maintain the flow rate to match the current, leading to a huge concentration gradient of lithium-ions in the electrolyte.

The electric potential formed by the difference in the lithium-ion concentration reduces the initial discharge voltage of the battery, thus resulting in a decrease in its effective capacity and discharge voltage platform. Introducing the buffer layer increases the migration rate of lithium-ions at the interface and facilitates their escape from the solid electrolyte and embedding into the cathode during the discharge process. For the batteries using liquid electrolytes, the high discharge rate of the battery is limited by the rate at which lithium-ions are disengaged and embedded into the electrode. For solid electrolyte batteries, the interface between the solid electrolyte and electrode is a direct factor of the limiting multiplier because of its complex structure and phase [34].

#### 3.3.2. Cycle Performance

The cycle characteristics directly affect the service life of the battery. A series of changes occurs in the battery, such as interface side reactions, dendrite growth, element diffusion, impurity phase formation, cracking, or pulverization, because of the volume change of the electrode after multiple charge and discharge cycles [35,36,37]. The battery cycling performance was measured with and without a buffer layer at a constant current of 3 µA (charging and discharging current are both 3 μA) and a cycling potential range of 3.2 to 4.2 V.

**Figure 4:**
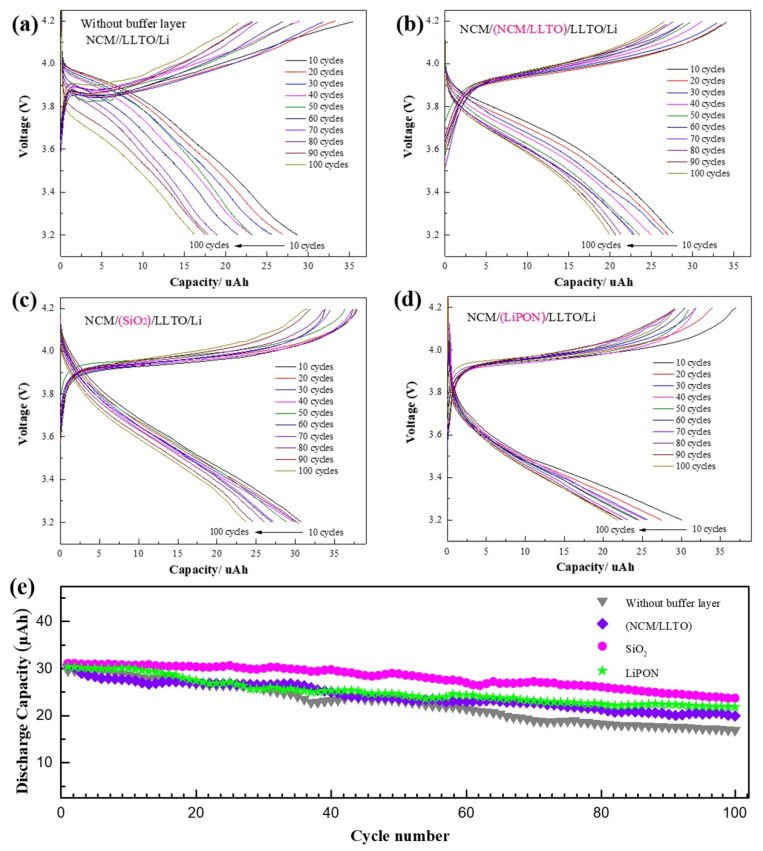
Cycle characteristics of battery with different buffer layer, capacity remained after 100 cycles at a 1 C rate between 3.2 and 4.2 V for battery with different interface structure: (**a**) NCM/LLTO, (**b**) NCM/(LLTO/NCM)/LLTO, (**c**) NCM/SiO_2_/LLTO, (**d**) NCM/LiPON/LLTO, (**e**) performance of first 100 cycles for cells with different buffer layer.

For all samples, the first 100 cycles were tested by constant current charging and discharging, and the constant current of 3 µA was equivalent to a discharge rate of 0.1 C. The capacity of the sample without a buffer layer reduced to 54% of the initial one after 100 cycles, and the initial discharge voltage and discharge platform of the battery decreased with the increasing number of cycles (Figure 4a). Using the buffer layer enhanced the cyclic stability of the battery capacity. The capacity of the sample with a homogeneous buffer layer decreased to 71% of the initial one after 100 cycles. The capacity of the samples with SiO_2_ and LiPON were maintained at 76% and 79%, respectively (Figure 4b–e). Insufficient contact between the electrode and the electrolyte occurs because of the volume change of the electrode and leads to a decrease in the effective contact area. Some electrode particles can no longer store lithium due to problems such as cracking, which results in the irreversible reduction of battery capacity. The compact buffer helps maintain the structural and chemical stability of the interface. It is also beneficial for reducing interface side effects, thus increasing the battery cycle performance [38].

#### 3.3.3. Cycle Performance of Battery with SiO_2_ Buffer Layer

**Figure 5:**
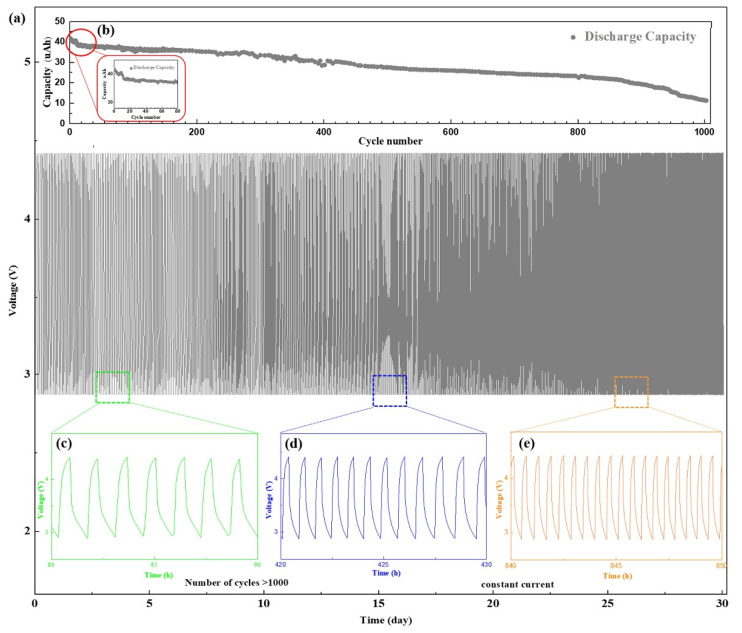
Cycling performance of the battery with NCM/SiO_2_/LLTO structure, which was measured with a constant current 3 µA with a cycling potential range from 2.8 to 4.2 V. (**a**) Charge/discharge curve of the battery (1000 cycles). (**b**) Cycle performance of the discharge capacity of the battery. Charging/discharging curves of the battery from the (**c**) 80th to 90th cycles (**d**) 420 to 430 cycles. (**e**) 840 to 850 cycles.

To study the impact of the buffer layer on the battery cycle life, we conducted 1000 deep discharge cycles for the sample with the SiO_2_ buffer layer for 32 days (Figure 5a–e). To increase the discharge depth, the cycling performance of the battery was tested by constant current method with a cycling potential range 2.8–4.2 V. The discharge current also increased from 3 µA (Figure 4e) to 40 µA. As shown in Figure 5b, the battery capacity decreased significantly after the first dozen cycles, presumably because of the gradual LiSiO_2_ formation from SiO_2_. The generation of LiSiO_2_ requires a certain amount of Li-ions [39], which led to a decrease in capacity. When the composition and thickness of the interface were relatively stable, the capacity of the battery decreased smoothly.

Figure 5c shows that the discharge current of 40 µA was equivalent to the discharge rate of approximately 1–1.5 C in the first 100 cycles. After 500 cycles, compared with the initial capacity, the battery capacity decayed to 65%. The discharge current was equivalent to the discharge rate of 2 C (Figure 5d), and the discharge platform decreased gradually with an increase in the discharge rate. After 900 cycles, the battery capacity reduced significantly. The sudden decrease in the battery performance was caused by various factors, such as the destruction of the interface structure or the cracking of the electrodes, and some irreversible phase changes at the interface [40]. The capacity of the battery decayed to 27.7% after 1000 cycles, and the discharge current was equal to the discharge rate of 3.6 C at this stage (Figure 5e). The battery cycle life depends on both the electrode and the interface, and any degradation in performance between them would shorten the cycle life. We demonstrated that optimizing the interface between the electrode and solid electrolyte by introducing a SiO_2_ buffer layer can improve the battery cycle life. For ASSBs, the number of cycles can reach as high as 800–1500 indicating the excellent performance of the batteries and their potential of practical applications [41].

## 4. Conclusions

In summary, we have optimized the ionic conductivity of LLTO thin films prepared by magnetron sputtering with annealing treatment. The highest ionic conductivity is achieved for the LLTO thin film annealed at 300 °C, which is 9.56 × 10^−6^ S/cm at room temperature and 7.78 × 10^−5^ S/cm at 120 °C. Two types of buffer layer structures were developed: homogeneous and heterogeneous. The batteries with a buffer layer revealed better capacity retention and rate characteristics than the pristine one during the charging/discharging in the potential range of 3.2–4.2 V. The compact SiO_2_ buffer layer ensures good contact with the electrode and solid electrolyte owing to an interface with good structural and chemical stability, and the cycle life of the batteries can exceed 1000 times. The extremely high cycling performance of the all-solid-state batteries with SiO_2_ buffer layer paves a way for their forthcoming commercial applications in energy storage fields.

## Figures and Tables

**Table 1:** Sample classification and the corresponding buffer layer.

Sample	A	B	C	D
Type	Without Buffer Layer	Homogeneous Buffer Layer	Heterogeneous Buffer Layer	Heterogeneous Buffer Layer
Buffer layer	/	Li_0.33_La_0.55_TiO_3_/ LiNi_0.5_Co_0.3_Mn_0.2_O_2_ (LLTO/NCM)	SiO_2_	LiPON
Battery structure	NCM/LLTO/Li	NCM/(LLTO/NCM)/LLTO/Li	NCM/SiO_2_/LLTO/Li	NCM/LiPON/LLTO/Li

## Data Availability

The data presented in this study are available on request from the corresponding author.
